# The membrane depolarization and increase intracellular calcium level produced by silver nanoclusters are responsible for bacterial death

**DOI:** 10.1038/s41598-021-00545-7

**Published:** 2021-11-03

**Authors:** Junior Bernardo Molina-Hernandez, Antonio Aceto, Tonino Bucciarelli, Domenico Paludi, Luca Valbonetti, Katiuscia Zilli, Luca Scotti, Clemencia Chaves-López

**Affiliations:** 1 Faculty of Bioscience and Technology for Food, Agriculture and Environment, University of Teramo, Teramo, Italy; 2 Department of Medical, Oral and Biotechnological Sciences, “G. d’Annunzio” University of Chieti-Pescara, Chieti, Italy; 3 Faculty of Veterinary Medicine, University of Teramo, Teramo, Italy; 4 Istituto Zooprofilattico Sperimentale dell’Abruzzo e del Molise Giuseppe Caporale, Teramo, Italy

**Keywords:** Microbiology, Nanoscience and technology

## Abstract

This work highlights how our silver ultra nanoclusters (ARGIRIUM-SUNc) hand-made synthesized, are very useful as a bactericide and anti-biofilm agent. The Argirium-SUNc effective antibacterial concentrations are very low (< 1 ppm) as compared to the corresponding values reported in the literature. Different bacterial defense mechanisms are observed dependent on ARGIRIUM-SUNc concentrations. Biochemical investigations (volatilome) have been performed to understand the pathways involved in cell death. By using fluorescence techniques and cell viability measurements we show, for the first time, that membrane depolarization and calcium intracellular level are both primary events in bacteria death. The ARGIRIUM-SUNc determined eradication of different biofilm at a concentration as low as 0.6 ppm. This suggests that the effect of the nanoparticles follows a common mechanism in different bacteria. It is highly probable that the chemical constitution of the crosslinks could be a key target in the disrupting mechanism of our nanoparticles. Since the biofilms and their constituents are essential for bacterial survival in contact with humans, the silver nanoparticles represent a logical target for new antibacterial treatments.

## Introduction

ESKAPE is an acronym that includes six pathogenic bacteria such as *Enterococcus faecium, Staphylococcus aureus, Klebsiella pneumoniae, Acinetobacter bamannii, Pseudomonas aeruginosa* and *Enterobacter* species, possessing multidrug resistance (MDR) and virulence^1^. In the last decades, MDR pathogens are an increasing public health concern around the world. The ESKAPE members have been isolated not only from the clinical environment but also from food industrial ones. In particular, Gram-negative bacteria such as *Enterobacter* species and *K. pneumoniae* have been isolated from foods and food contact surfaces^2–8^. The persistence of MDR microorganisms in clinical and foods environment is favored by biofilm formation and it is considered a real threat to human health^9^. This structure confers different advantages to bacteria, such as interaction and cooperation, nutrient and enzyme entrapment, heterogeneous habitat, and tolerance to antimicrobial agents^10^. Growth in biofilms enhances not only the survival of bacterial populations but also genetic transfer between bacteria and it could be responsible for the persistence and transmission of antimicrobial resistance genes^11^. Several strategies have been used to prevent or eliminate biofilm production from the different environments among them: properly executed cleaning and disinfection with appropriate detergents, disinfectants, and enzymes; control of environmental factors and quorum sensing inhibitors. However, there is increasing evidence of co-resistance and cross-resistance between a range of clinically important antibiotics and disinfectants^12^. Thus, in the last years, the use of nanotechnology to produce antimicrobial compounds has been proposed. In particular, silver nanoparticles (AgNPs) have shown to be promising in the wounds, the coating of other surfaces, in food packages to extend the food shelf-life and to maintain its quality^13^. AgNPs have been shown strong biocidal effects on a broad spectrum of bacteria and fungi^14,15^. In addition, AgNPs are able to diffuse across the biofilm EPS network before releasing Ag^+^ ions, leading to a high kill rate of the bacteria in the biofilm lower layers^16^. Another hypothesis would be that silver binds non-specifically to a wide variety of targets, perturbing simultaneously many aspects of the cell metabolism and leading to its death^17^. In addition, it has been evidenced that some physicochemical properties of AgNPs as shape, size, chemical composition, and surface functional groups have direct influences on their interaction with the cell membrane^18^. The AgNPs induced structural changes in phospholipid may lead to the loss of amphiphilic properties, membrane disrupting and cell leaking^19^. Perturbation of membrane integrity by nanoparticle-membrane electrostatic interactions has been suggested to cause cellular dysfunction in an acute manner^20^. Therefore, in the present study for the first time, we evaluated the activity of a novel ARGIRIUM-SUNCs (Argirium Silver Ultra Nano Clusters) against multidrug-resistant *Enterobacter roggenkampii, Enterobacter hormaechei* two members of *E. cloacae* complex, and *K. pneumoniae* strains characterized by the capability to form biofilm. In addition, in the present work, we used several approaches, which include a combination of different metabolic and cellular bacterial analyses to better understand the events that are plays a dominant role of the bactericidal action of the ARGIRIUM-SUNCs in the above-mentioned species. We determined the biofilm eradication, growth inhibition, the time-kill kinetics, the change of cell membrane potential (ΔΨ), as well as metabolic changes such us bacterial reactive oxygen species (ROS) production, and glutathione depletion, to evaluated the toxic effect of ARGIRIUM-SUNCs.

## Materials and methods

### Synthesis and characterization of ARGIRIUM-SUNCs

ARGIRIUM-SUNCs were electrochemically synthesized by an improved synthetic protocol in ultra-pure water without stabilizing agents or other chemical components as previously reported^21^. The point-of-novelty is an “ultra-small nanoparticles” size without changing the complex set of chemical–physical properties (Z-Potential), plasmonic UV–Vis absorbance, concentration, stability in acid pH. The method is protected by European Patent (EP-18181873.3). ARGIRIUM-SUNc solution was yellow with an absorbance maximum at 410 nm, odorless, pH of 7–8, and characterized by good stability (> 1 year). No change in absorbance and λ_max_ value was observed after 1 h of incubation of SUNCs at pH 3.5 in 0.01 M acetate buffer^22^. A drastic decrease of the plasmonic spectrum absorbance, without change of the λ_max_, was observed after nitric acid treatment (0.1 M, pH < 1) for 10 min at 25 °C^22^.The complete disappearance of the ARGIRIUM-SUNCs UV–Vis spectrum is obtained only after 60 min of nitric acid (0.1 M final concentration) reaction at 60 °C^23^. After each synthesis, larger nano clusters were removed by flushing the solution through 0.1 μm syringe filter devices (Whatman CYCLPR) and subsequent centrifugation at 13.023×*g* for 15 min. SUNCs were characterized by Transmission Electron Microscopy (TEM-Jeol Japan) in terms of concentration, shape, and size determination (75 kV ZEISS 109 equipped with Gatan-Orius SC200W-Model 830.10 W TEM CCD Camera). Particles concentration was taken at 75 kV after evaporation of a diluted (1:5) drop ARGIRIUM-SUNCs solution on 300 mesh formvar coated nickel grids, and confirmed by ions selective electrode technique^24^ (ISE). Evidence of non-spherical shape was reported by TEM at × 250,000 magnification, and the images elaborated with ImageJ software (ImageJ bundled with Java 1.8.0_172 https://imagej.nih.gov/ij/download.html). Particles numbers and distribution were calculated using a statistical software Origin ver 9.0; their good stability in the time (> 1 year) was also verified. The morphology as well as the elemental composition of the particles were analyzed by Scanning Electron Microscopy (SEM) using a Phenom xl (Thermo Fisher Scientific, USA) equipped with BDS, SED and EDS detectors (15 kW of acceleration voltages under high vacuum level).

### Bacterial strains and inoculum

The bacteria used in present study were isolated from the foods surface contact and belong to the culture collection of the Faculty of Bioscience and Technology for Food, Agriculture and Environment, University of Teramo (Italy). Cultures of strains were grown on Tryptic Soy Broth (TSB, Oxoid, Italy) at 37 °C and stored at − 20 °C on Plate Count Agar (PCA) Petri dishes. The strain cultures were maintained at − 80 °C in cryovials, containing glycerol, 20% v/v, (Sigma). Bacterial strains, from stock cultures, were grown overnight in TSB, at 37 °C. After 18 h of incubation, one mL of bacterial suspension was transfered into fresh Brain Heart Infusion Broth (BHI) supplemented with 0.2% of glucose and incubated at 37 °C for 18 h. Successively, strains were harvested by centrifugation and standardized at 0.1 of absorbance (about 3 × 10^7^ CFU/mL), as reported by Ref.^10^. The inoculum was confirmed by plate counts in Violet Red Bile Glucose Agar (BRVGA) (Oxoid), incubated at 37 °C for 24 h. The strains were characterized for their capability to form biofilm following the method used by Ref.^10^, and the antibiotic resistance was assessed by using the automated system VITEK^®^ 2 system, (BioMèrieux, Marcy L’Etoile, France) according to the manufacturer’s instructions and an Agar Plate Antibiotic Disk Diffusion (Kirbie–Bauer) method according to Clinical and Laboratory Standards Institute (CLSI guideline).

### Antibacterial activity of ARGIRIUM-SUNCs and bacterial growth dynamics

Bacteria growth kinetics of the cells exposed to ARGIRIUM-SUNCs were assessed by the broth microdilution method. The experiment was done in 96-well polystyrene microplates. Also in this case, each strain was tested in 5 replicates and the experiment was repeated twice. The standardized inoculum (1 × 10^7^ CFU/mL) was exposed to a different ARGIRIUM-SUNCs concentration (0, 0.625, 1.25, 2.5, 3.0, 4.0, 5 and 10 ppm) and incubated at 37 °C. The growth increase was measured by using of OmniLog instrument (Biolog INC., Hayward, USA). The values were expressed as mean of five replicated growth curves.

Data obtained were also analyzed over time according to the Gompertz equation modified by Ref.^25^:

$$ {\text{Y}}\left( {\text{t}} \right) \, = {\text{ b1 }} \times {\text{ exp }}\left( { - {\text{exp }}\left( {\left( {{\text{b2 }} \times { 2}.{7182}/{\text{b1}}} \right) \, \times \, \left( {{\text{b3}} - {\text{t}}} \right) \, + { 1}} \right)} \right), $$

where **Y** is the number of CFU at each time of the experiment, **b1** is the maximum CFU achieved during stationary phase, **b2** is the maximum growth rate (1/h) and, **b3** is the lag phase (hours) and **t** is the time. The data collected were the means of five independent repetitions.

### Time kill kinetics

Based on the results of growth inactivation, and biofilm formation, two strains that showed the major resistance (*E. roggenkampii* ECA 4) and major sensitivity (*K. pneumoniae* ECA 3-2), to ARGIRIUM-SUNCs, were further studied for the time-kill kinetics (TKK) in BHI broth as reported by Ref.^26^.

### Biofilm production and eradication

The biofilm-forming ability and quantification as well as the biofilm eradication were performed following the method used by Rossi et al.^7,10,27^. In our case after 24 h of biofilm formation samples were exposed to the following concentration of ARGIRIUM-SUNCs: 0.625, 1.25; 2.5; 5 and 10 ppm.

### Cell viability analysis

Confocal laser scanning microscopy was used to quantify the number of live cells and dead cells using a mixture of CFDA (carboxyfluorescein diacetate) and propidium iodide (PI). While green fluorescent dye CFDA permeated both intact and damaged cell membranes, the red fluorescent dye propidium iodide (PI) entered the only cells with significant membrane damage.

Bacteria suspensions of *E. roggenkampii* ECA 4 or *K. pneumoniae* ECA 3-2 were previously standardized to 0.1 OD (approximately 1 × 10^7^ CFU/mL). Successively bacteria were inoculated in BHI and exposed to ARGIRIUM-SUNCs at MIC values obtained previously (5 ppm), for 10 h. Isopropanol at 70% v/v for 1 was used as positive control^28^. After that, samples were stained with CFDA and incubated 37 °C for 30 min and further with PI and left 30 min at 18 °C. Cells were washed with phosphate buffer saline (PBS) after staining ensuring the permanence of the pellet, finally, 10 μL of bacteria were deposited in polyresin cover slides and observed with Nikon A1R confocal imaging system (Nikon Corp., Tokyo, Japan) as previously reported.

### Membrane depolarization assessment

In order to evidence the membrane depolarization, bacteria cells were grown and standardized as reported above, and re-suspended in phosphate-buffered saline (PBS), and successively treated with 5 ppm of ARGIRIUM-SUNCs or 250 μL of isopropanol-TSB (70% V/V) as positive control and 250 μL of TSB were as negative control. Samples were incubated for 1 h. Then, one ml of DiBAC4 Bis-(1,3-Dibutylbarbituric Acid Trimethine Oxonol) (Molecular Probes, Eugene, OR, USA) staining ethanol solution (1 mg/mL) was added. After incubation for 15 min at room temperature, the samples were washed twice in PBS. Finally, in order to observe the membrane depolarization, we used a Nikon A1R confocal imaging system (Nikon Corp., Tokyo, Japan), controlled by the Nikon NIS Elements interface, equipped with a Plan Apo k 100 9 Oil objective (numerical aperture: 14; Refractive Index: 1515). The specific function Nikon A1 Piezo Z Drive, detector Galvano, pinhole size of 690 µm, Z-step: 015 µm, with excitation of 488 nm and emission of 515 nm.

### Glutathione assay

The intracellular glutathione was determined in bacteria cells treated with 5 ppm of ARGIRIUM-SUNCs at different times of incubation (0, 2, and 10 h). In all cases, cells were washed three times in PBS by centrifugation at 600×*g* to obtain a packed cell pellet, concentration was adjusted to 10^7^ CFU/mL. Cell extracts and glutathione quantification were obtained according to the producers of the Glutathione Assay Kit (Catalog Number CS0260). The absorbance of the reaction mixture was monitored at 415 nm with a microtiter Multimode Plate Reader ENSPIRE (Perkin Elmer, USA) sequent the standard curves, and deproteinated supernatants were analyzed.

### Measurement of intracellular *ROS*

Intracellular ROS accumulation was assessed using 2′,7′-dichloro-dihydro-fluorescein diacetate (H_2_DCFDA; Molecular Probes). The experiment was performed in 96-well polystyrene microplates. Bacteria cells concentration was adjusted to 10^7^ CFU/mL and incubated in the presence of 5 ppm of ARGIRIUM-SUNCs for 2 h. After that cell were washed with PBS and re-suspended, then 10 µM H_2_DCFDA were added and incubated for 1 h. Subsequently stained cells were washed with PBS and the fluorescence was detected by measuring at excitation and emission wavelengths of 485 nm and 535 nm with a microliter Multimode Plate Reader ENSPIRE (Perkin Elmer, USA).

### Measurement of intracellular calcium levels (Ca^+2^)

Calcium levels were measured with fura-2 acetoxymethyl ester (AM) (Molecular Probes) following the methodology reported by Refs.^29,30^ with some modifications. Bacterial cells 1 × 10^7^ CFU/mL were incubated with 5 ppm of ARGIRIUM-SUNCs or 30% v/v of H_2_O_2_ (control samples) for 2 h at 37 °C. After that, cells were washed twice in Krebs Buffer 7 Fura-2AM fluorescence was recorded at 340/380 nm excitation and 512 nm emission. The peak amplitude of Fura-2AM fluorescence (ratio at 340/380 nm) was used to evaluate ER calcium levels.

### Volatile compounds production after stress of ARGIRIUM-SUNCs

This part of the work was aimed to study the effect of sub-lethal concentrations of ARGIRIUM-SUNCs on the bacteria metabolism. Thus, the strains showing the highest sensitiveness against ARGIRIUM-SUNCs (*K. pneumoniae* ECA 3-2) and highest resistance (*E. roggenkampii* ECA 4), were analyzed after exposure at 0.625 and 1.25 ppm. An 100 μL of a 24 h pre-culture of the strains (obtained as above described) were inoculated in 5 mL of TSB broth using vials of 20 mL. After closing hermetically the vials were incubated at 37 °C in dynamic conditions (100 rpm) up to 48 h. Six samples for each concentration and for each strain were prepared and non-inoculated vials containing only TSB were used as control. Three samples were analyzed each 24 h for the determination of volatile compounds (VOCs) following the methodology proposed by Ref.^31^. An Agilent Hewlett Packard 6890 GC gas chromatograph equipped with a MS detector 5970 MSD (Hewlett Packard, Geneva, Switzerland) was used for peak separation and detection. A fused silica capillary column was a CP Wax 52 CB (50 m × 0·32 mm—Chrompack—Middelburg, the Netherlands), coated with polyethylene glycol (film thickness 1·2 μm) as stationary phase was used. The injector and FID temperature was 250 °C; detector temperature 220 °C; carrier gas (He) flow rate, 1 mL/min.

### Statistical analysis

The data are expressed as means ± standard deviations. Data were evaluated by analysis of variance (ANOVA) and compared by 95% Tukey’s HSD test, using Statistica 13.5 software (TIBCO, Tulsa, OK, USA).

## Results and discussion

### Characterization of ARGIRIUM-SUNCs

**Figure 1:**
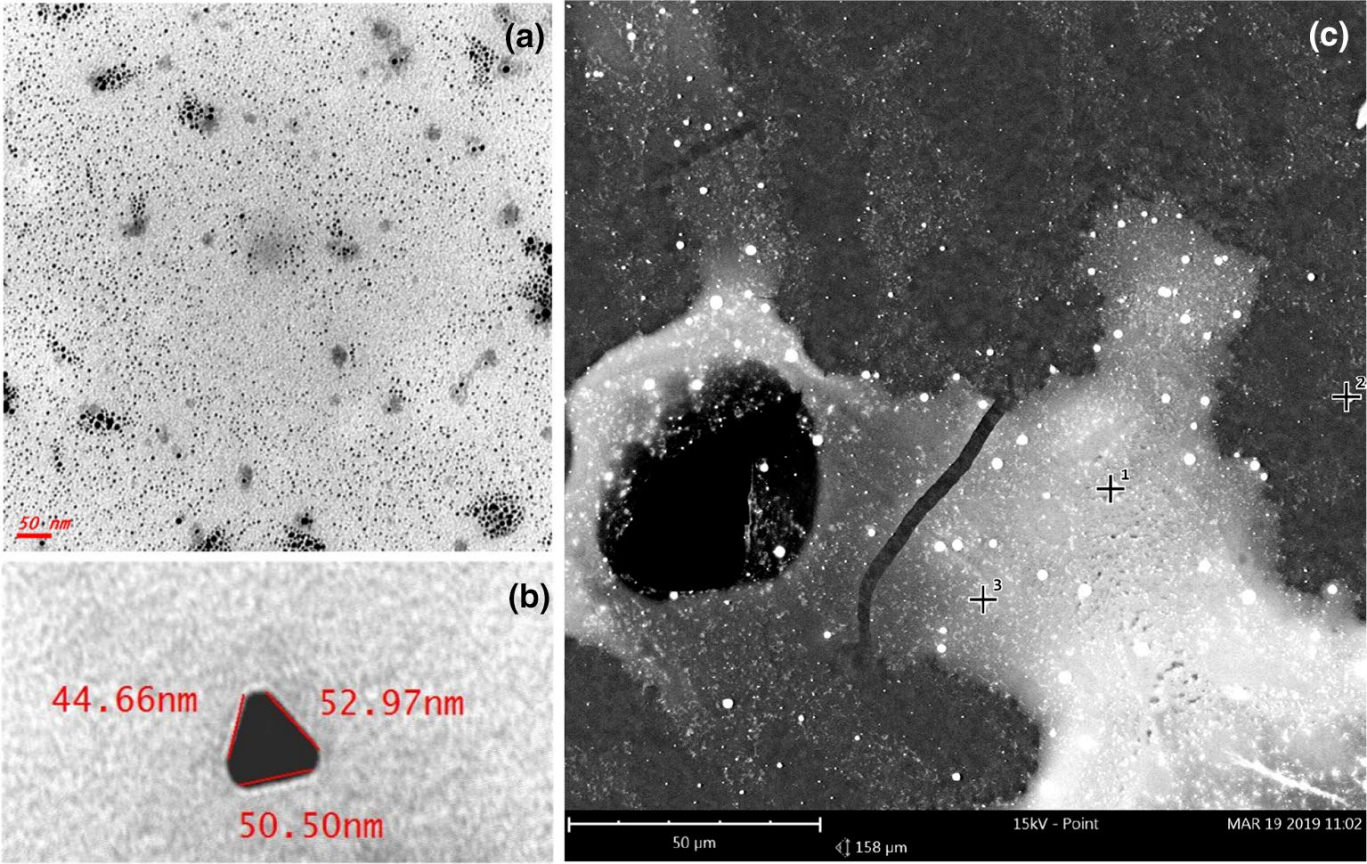
(**a**) TEM of filtered SUNc in ultra-pure water (0.22 μm). (**b**) A magnification SUNc TEM image not filtrated. (**c**) Scanning electron microscopy analysis of SUNCs. Magnification: × 85,000 (scale bar: 10 μm–15 kV). Spots 1, 2, and 3 revealed different distribution of elements in the sample-different phases according to the SEM elemental analysis.

In a typical preparation procedure, the ARGIRIUM-SUNCs final concentration, as determined by ion-selective-electrode (ISE) in aqueous solution at pH 7.8, was about 20 mg/L. At the analysis of the TEM, nanoclusters resulted non spherical with an average size particularly small of 1.79 nm ± 1.004 (Fig. 1). The statistical analysis is reported as Supplementary Material (Fig. S1). The presence of plasmonic resonance spectrum (λ max at 410 nm, UV) and data obtained by SEM analysis confirm that silver metal (Ag^0^) was predominant in our formulation (Fig. 1c). As observed at SEM a second abundant element was oxygen suggesting the possibility for the presence of Ag at different oxidation states (Ag^0^/Ag^n+^). These chemical-physical properties substantially distinguish our nanoparticles from any other formulation and can explain the higher antibacterial efficacy. Another not negligible feature is that our nanoclusters, unlike of many phytogenic or chemically synthesized AgNPs are stable for several months and their synthesis is made in water without organic/inorganic contaminants/stabilizer^32,33^. ARGIRIUM-SUNc formulation resulted stable also at acidic condition (pH < 4). These properties are particularly relevant in terms of their potential use as new drugs, both alone or linked to traditional antibiotics.

### Characterization of the strains

Biofilm formation in medical and food environments is of special interest because they can act as a source of microbial contamination and can contribute to food spoilage or to the transmission of the disease. In our experiment, the quantification of the biofilm formed after 24 h is shown in Table 1. In general, the biofilm production was strain-dependent with *E. roggenkampii* ECA 4 and *E. hormaechei* ECA 23/3 classified as moderate biofilm producers while *K. pneumoniae* ECA 3-2 and ECA 20/3 strains as weak biofilm producers. The moderate capacity of the *Enterobacter cloacae* complex has been reported, however, no data are available in literature for *E. roggenkampii* which is a recently described species that can cause multi-site infection (e.g. blood, urine, feces, and body fluids)^33^. On the other hand, studying the biofilm production of 9 strains of *E. hormaechei* from clinical origin reported that they formed a moderate biofilm^34^. Other studies have been evidenced the capability of *K. pneumoniae* to produce weak and moderate biofilm. As depict (Table S1-Supplementary Materials), the strains of *K. pneumoniae* and *E. roggenkampi* used in this study, showed antibiotic resistance against macrolides (Oleandomycin, Clindamycin, Erythromicin and Spiramycin), β-lactams (Oxacillin, Amoxicillin, and Ceforoxime), polypeptide (Bacitracin), oxazolidinones (Linezolid), and the cephalosporin cephalothin. The antibiotic resistance of *K. pneumoniae* towards penicillin, cephalosporin, fluoroquinolone, aminoglycoside, sulfonamide as well as against third-generation cephalosporins, and aminoglycosides, has been reported^35^. It has been also reported that the MDR of *K. Pneumoniae* to b-lactams and third- and fourth-generation cephalosporins, is due to plasmid-encoded extended-spectrum beta-lactamases (ESBLs). On the other hand, some studies have been evidenced a positive correlation between antibiotic resistance profile and biofilm-forming ability in *K. pneumoniae*^36^. In this context, restricted penetration of antibiotics through biofilms may occur in cases where the antibiotics bind to components of the biofilm matrix or the bacterial membranes such as extracellular DNA.

**Table 1:** Production of biofilm after 24 h of exposure to Arg-SUNCs at 37 °C.

Strain	Optical density (OD_590_)	Biofilm grade
*Klebsiella pneumoniae* **ECA3-2**(*)	0.889 ± 0.033	Weak
*Klebsiella pneumoniae* **ECA20-3**(*)	0.732 ± 0.017	Weak
*Enterobacter roggenkampii ***ECA4**(*)	1.587 ± 0.017	Moderate
*Enterobacter hormaechei* **ECA23-3**(*)	1.441 ± 0.031	Moderate

The absorbance at 590 nm [optical density (OD)_590_] was measured, and the strains were grouped into: OD_590_ < 0.1, non-producers (NP); OD_590_ = 0.1–1.0, weak producers (WP); OD_590_ = 1.1–3.0, moderate producers (MP); and OD_590_ > 3.0, strong producers (SP). (*) are conventional names of strains.

### Effects of ARGIRIUM-SUNCs on growth inhibition of the strains

In order to establish the survival/inactivation kinetics of the strains, they were inoculated in TSB and successful exposed to increasing ARGIRIUM-SUNCs concentrations. Data obtained from the five replications for each isolate showed that, nanomaterials were effective against all MRD strains. In particular, the nanomaterials had effect at concentrations > 2.5 mg/mL, and its efficacy increased with the concentration reaching the MIC at 5 mg/mL. Data obtained were analyzed according to the modified Gompertz equation in order to obtain the growth parameters lag phase (λ), maximum growth rate (*μ*_*max*_) and maximum density *A*_*max*_ of cells. The predicted curves fitted well with the experimental points and their regression coefficients were between 0.990 and 0.996. As observed in Table 2, with the increase of the ARGIRIUM-SUNCs, except to *E. roggenkampii* ECA4, the other strains showed a lag phase extension of about 10 h. In addition, a decrease of the maximum growth rate was achieved with the increase of the ARGIRIUM-SUNCs concentration from 3 to 4 ppm. To the best of our knowledge, we are unaware of any previous studies investigating the antibacterial activity of ARGIRIUM-SUNCs against *E. roggenkampii* and *K. pneumoniae*.

**Table 2:** Influence of Arg-SUNCs concentration, on the growth parameters of *K. pneumoniae* (ECA3-2), (ECA20-3) *E. roggenkampii* (ECA 4) and *E. hormaechei* (ECA23-3) in TSB Medium, calculated using modified Gompertz Equation.

Gompertz parameters																
*K. pneumoniae* (ECA3-2)					*K. pneumoniae* (ECA20-3)				*E. roggenkampii* (ECA4)				*E. hormaechei* (ECA23-3)			
Argirium-SUNCs (ppm)	*λ*	*µ*_*max*_	*A*_*max*_	R	*λ*	*µ*_*max*_	*A*_*max*_	R	*λ*	*µ*_*max*_	*A*_*max*_	R	*λ*	*µ*_*max*_	*A*_*max*_	R
Control	4.12 ± 0.01^a^	0.23 ± .01^a^	1.46 ± 0.02^a^	0.992	4.34 ± 0.02^a^	0.22 ± 0.01^a^	1.58 ± 0.01^a^	0.992	4.25 ± 0.07^a^	0.19 ± 0.07^a^	1.48 ± 0.07^a^	0.996	5.2 ± 0.02^a^	0.19 ± 0.02^a^	1.48 ± 0.2^a^	0.993
0.625	12.9 ± 0.01^b^	0.17 ± 0.01^b^	1.47 ± 0.01^a^	0.991	14.0 ± 0.03^b^	0.14 ± 0.03^a^	1.46 ± 0.02^a^	0.994	4.25 ± 0.02^a^	0.21 ± 0.02^a^	1.49 ± 0.02^a^	0.994	14.1 ± 0.09^b^	0.15 ± 0.09^b^	1.48 ± 0.3^a^	0.994
1.25	13.2 ± 0.01^b^	0.21 ± .02^a^	1.47 ± 0.01^a^	0.993	14.2 ± 0.03^b^	0.14 ± 0.03^a^	1.48 ± 0.02^a^	0.992	4.40 ± 0.04^a^	0.20 ± 0.02^a^	1.21 ± 0.03^b^	0.993	14.1 ± 0.30^b^	0.16 ± 0.2^b^	1.48 ± 0.1^a^	0.993
2.5	13.5 ± 0.03^b^	0.29 ± 0.01^c^	1.45 ± 0.02^a^	0.993	14.0 ± 0.03^b^	0.17 ± 0.03^a^	1.48 ± 0.02^a^	0.995	4.51 ± 0.06^b^	0.19 ± 0.06^a^	1.24 ± 0.06^b^	0.995	14.3 ± 0.03^b^	0.17 ± 0.02^ab^	1.47 ± 0.2^a^	0.991
3	13.2 ± 0.01^b^	0.04 ± 0.02^d^	1.40 ± 0.01^b^	0.994	14.1 ± 0.02^c^	0.05 ± 0.02^b^	1.47 ± 0.01^b^	0.992	4.47 ± 0.05^b^	0.02 ± 0.05^b^	1.26 ± 0.04^b^	0.991	14.1 ± 0.01^b^	0.06 ± 0.01^c^	1.48 ± 0.3^a^	0.991
4	13.5 ± 0.01^b^	0.04 ± 0.03^d^	1.46 ± 0.02^a^	0.992	14.2 ± 0.01^c^	0.05 ± 0.01^b^	1.43 ± 0.01^b^	0.996	3.92 ± 0.01^c^	0.02 ± 0.02^b^	1.23 ± 0.01^b^	0.990	14.1 ± 0.04^b^	0.06 ± 0.03^c^	1.43 ± 0.2^a^	0.991
5	N.G.	N.G.	N.G.	N.G.	N.G.	N.G.	N.G.	N.G.	N.G.	N.G.	N.G.	N.G.	N.G.	N.G.	N.G.	N.G.
10	N.G.	N.G.	N.G.	N.G.	N.G.	N.G.	N.G.	N.G.	N.G.	N.G.	N.G.	N.G.	N.G.	N.G.	N.G.	N.G.

Mean and standard deviation of five repetitions. Different lowercase letters (a, b, c) indicate significant differences (p ≤ 0.05).

*A*_*max*_ maximum absorbance (expressed as DO units) during stationary phase, *μmax* maximum growth rate (1/h), *λ* lag phase (h), *N.G*. not grown.

### Time kill kinetics

**Figure 2:**
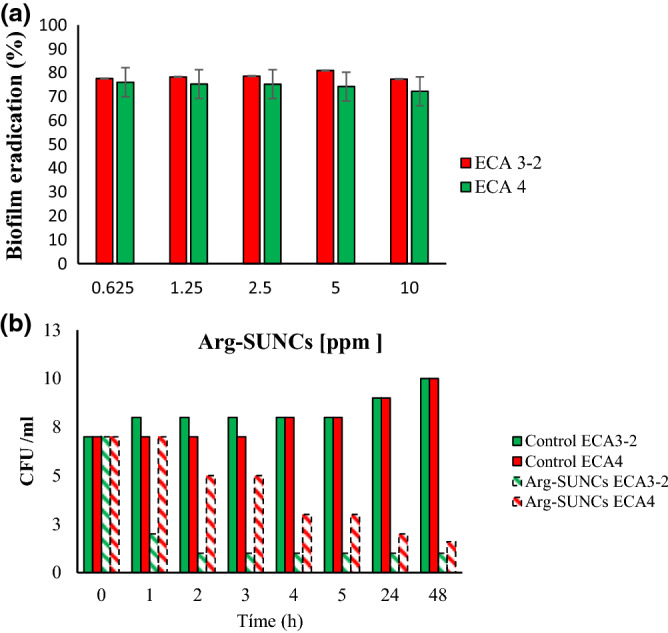
(**a**) Percentage of eradication of biofilm formed by *K. pneumoniae* (ECA3-2) and *E. roggenkampii* (ECA4) and by using different concentration of Arg-SUNCs. (**b**) Time-kill kinetics for the strain ECA4 and ECA3-2 treated with 5 ppm of Arg-SUNCs.

In order to evaluate the cell eradication potential of the ARGIRIUM-SUNCs, the strains ECA4 and ECA 3-2, selected for the capability to form a moderate biofilm and weak biofilm respectively, were subjected to a TKK assay and the kinetics of inactivation were monitored for 48 h. In general, different patterns of inhibition were showed by the two strains (Fig. 2b), as evidenced the less biofilm producer (ECA 3-2) was the most sensible strain, while the most biofilm producer was the most resistant strain. In particular, for the strain *K. pneumoniae* ECA 3-2 we observed a significant strong reduction (p > 0.05) of about 5 Log (UFC/ml) after the first hour of exposure to ARGIRIUM-SUNCs, a continued exposure determined a further reduction of 1 Log CFU, that remained constant until the 48 h of treatment, indicating a bacteriostatic effect.

On the contrary *E. roggenkampii* ECA 4, showed a reduction of 5 log CFU only after 24 h of exposure to the nanomaterial. Also, in this case the activity of the of Argirium^®^ SUNCs at 10 µg/mL was bacteriostatic. These differences in sensitivity could be attributed to the diverse surface properties (e.g., hydrophobicity) among *K. pneumoniae* ECA 3-2 and *E. roggenkampii* ECA 4, which would generate different interactions between cells and ARGIRIUM-SUNCs. In fact, it is well known that in a liquid culture the presence of both hydrophilic and hydrophobic cells is common, in addition environmental conditions can induce a switch of the microbial phenotype between hydrophobic and hydrophilic.

We would like to point out that *K. pneumoniae* ECA3-2 and *E. roggenkampii* ECA4 probably possessed different hydrophobicity, and this could have changed the surface charges of strains, which would certainly modify the interactions with ARGIRIUM-SUNCs.

In this context, Gram negative bacteria possess a membrane outside the peptidoglycan layer which provides a selective barrier against harmful agents. The interaction of ARGIRIUM-SUNCs with lipopolysaccharide (LPS) present in the outer membrane of *E. coli* has been observed with the O-antigen part of LPS as responsible for interaction of the nanoparticles through hydrogen bonding surface, inducing the LPS release and damaging the bacterial outer membrane. These actions permit the diffusion of ARGIRIUM-SUNCs into periplasm where they act similarly to Ag^+^ ions, interacting with SH-groups containing enzymes due to the very high affinity of Ag^+^ ions toward sulfur-containing groups^37^. Thus, it could be probably that the same interaction occurs in *K. pneumoniae* ECA 3-2 and *E. roggenkampii* ECA4 strains here studied. In addition, of ARGIRIUM-SUNCs can anchor to the bacterial cell wall and consequently penetrate this structure causing physical changes in the bacterial membrane, like depolarization of the cell membrane, which may result into cellular contents leakage and bacterial death^38^.

### Biofilm eradication

Biofilm eradication represents one of the biggest challenges to reduce the risk associated with microbial persistence of the pathogens in medical and foods-contact surfaces. Thus, there is a necessity of alternatives to traditional antibiotics that directly inhibit and/or eradicate the biofilms. To observe the effect of the ARGIRIUM-SUNCs on the biofilm eradication, we treated the 48 h biofilm formed by *K. pneumoniae* ECA 3-2 and *E. roggenkampii* ECA4 with different concentration of the nano compounds. As shown in Fig. 2a, we observed a strong biofilm eradication with low concentration of ARGIRIUM-SUNCs that reached values of 72.2% for *E. roggenkampii* ECA4 and 80.93% for *K. pneumoniae* ECA3-2 when compared with untreated control. It should be noted that this activity was not dose dependent. Rajivgandi et al. suggested that AgNPs inhibit the biofilm formation of *K. pneumoniae* through damaging the extracellular polysaccharide matrix which acts as a protective layer around the bacterial colonies. On the other hand, proteins and enzymes are also critical components of the biofilm matrix, thus it could be probable the Ag^+^ ions attached to cysteine residues of protein and collapsed the extracellular polysaccharide matrix in biofilm.

### Cell viability

**Figure 3:**
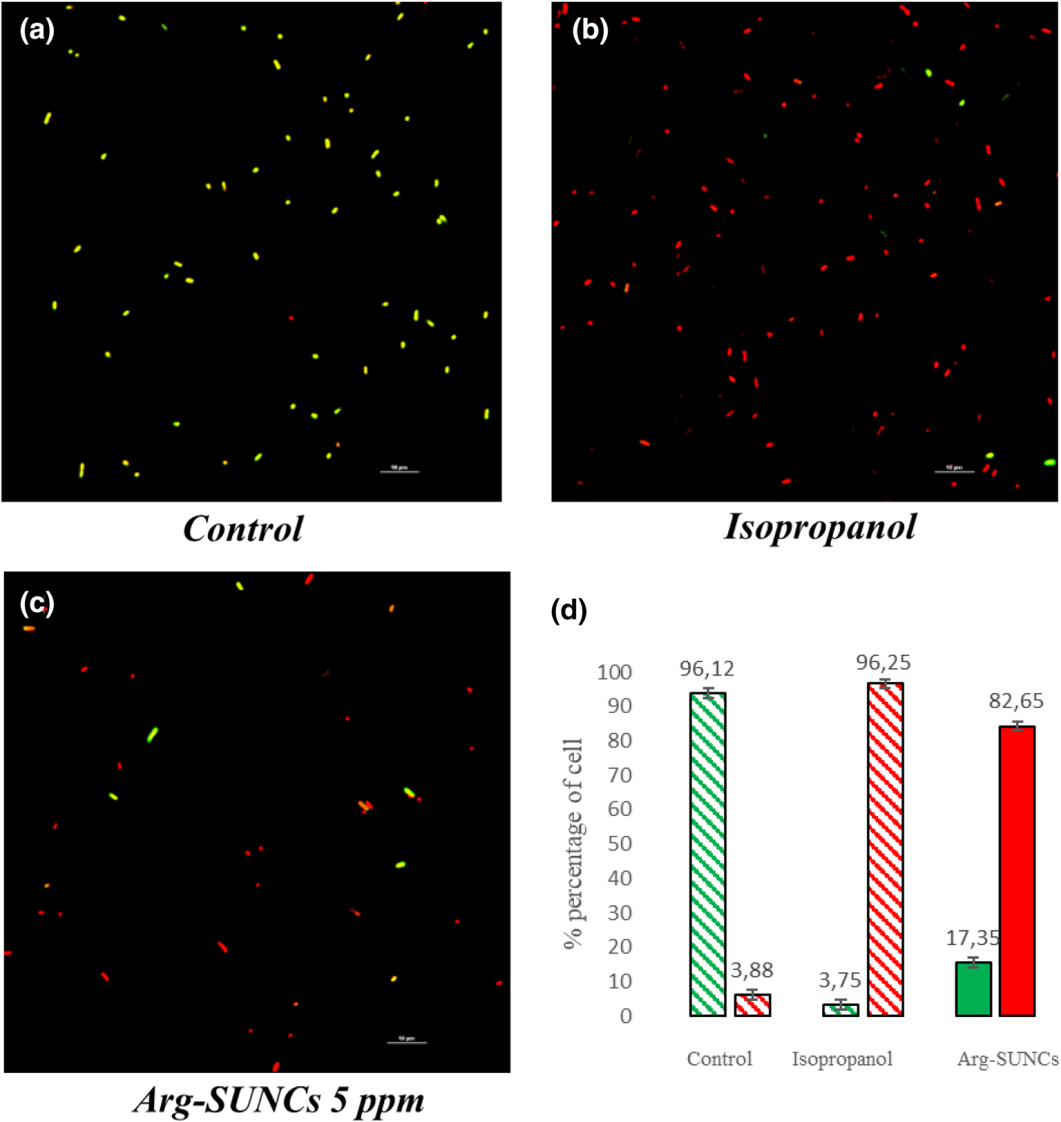
Live and death cells of *K. pneumoniae* (ECA3-2) visualized by fluorescence microscopy after staining with CFDA (carboxyfluorescein diacetate) and PI (propidium iodide). (**a**) Control, (**b**) isopropanol, (**c**) treated with 5 ppm of SUNCs (10 h), (**d**) quantification of live and death as (green) intact bacteria structure; (red) damaged bacteria structure.

In order to visualize and quantify the effect of ARGIRIUM-SUNCs, the strains were exposed for ten hours to 10 ppm of the nano-compounds. Propidium iodide (PI) and Dichlorofluorecin diacetate (CFDA) probes were used to ascertain the effects of the nanomaterial on the viability of the bacteria. Viable cells were those that did not show any damage at the membrane level and were directly visualized with the green fluorescence of the CFDA probe. Dead cells (with membrane damage) were visualized with the red fluorescence of propidium iodide probe. In general, the two tested strains showed similar behaviors, as example we here reported the effect on *K. pneumoniae* ECA3-2 (Fig. 3). As observed, in the control group, bacteria (Fig. 3a) bind dye CFDA and visible green fluorescent color indicates a health and viable cells. After ARGIRIUM-SUNCs treatment (Fig. 3c), visible fluorescent red color was observed in approximately 82% (Fig. 3d) of the cells suggesting a cell wall and membrane damage that resulted in cell death. Figure 3b showed cells treated with isopropanol (positive control for dead cells).

### The change of cell membrane potential (ΔΨ) and intracellular Ca^2+^

**Figure 4:**
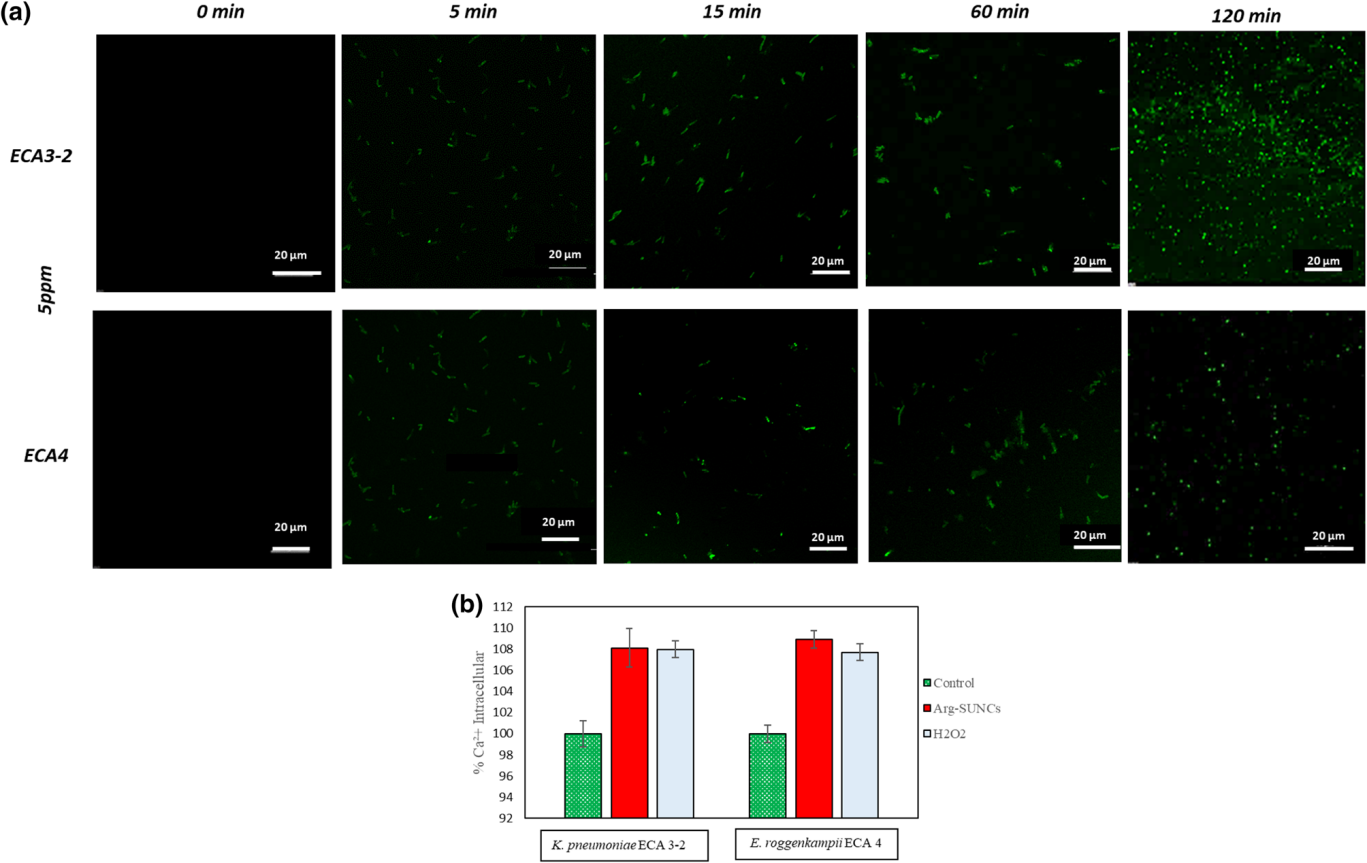
Effect of Arg-SUNCs on membrane depolarization (**a**) and intracellular calcium level (**b**). Cell were incubated for 0, 5, 15, 60 and 120 min with Arg-SUNCs then stained with DiBAC4. Calcium level, was measured with using Fura-2AM. The data represents the average standard deviation and p values.

Several factors influence bacterial cell viability and among them the membrane integrity, enzyme activity, membrane potential (ΔΨ) and nucleic acid integrity. To get direct evidence that ARGIRIUM-SUNCs are responsible for the change in ΔΨ, involved in cell energy production, membrane integrity and bacteria physiological state^39^, we used the green-fluorescent DiBAC_4_. This label enters the cell linking to intracellular proteins when the membrane potential is lost. As evidenced in Fig. 4a, a decrease in membrane potential was observed in treated cells with the ARGIRIUM-SUNCs. A major membrane depolarization was observed in *K. pneumoniae* ECA 3-2 as compared to *E. roggenkampii* ECA 4, suggesting a different response of each cellular membrane to the nanomaterial. Mechanistic studies suggest nanoparticles can alter the biological system of interest in subtle, yet important, ways^40^. The exposure of silver nanoparticles to bacteria produces structural changes in the cell membrane blocking the transport channels^41^. To determine whether increased intracellular calcium levels interfere with *K. pneumoniae* ECA 3-2 and *E. roggenkampii* ECA4 death, calcium levels were measured with fura-2 AM. As shown in Fig. 4b the exposure of the strains to 5 ppm of ARGIRIUM-SUNCs for 2 h, resulted in a significant overload of intracellular free calcium. These results are similar to those reported by Lew et al.^28^, which observed an increase of free calcium levels in *E. coli* cells after treatment with silver nanoparticles. It is very likely that the cell membrane depolarization could alter the channel properties thereby leading to the influx of intracellular Ca^2+^. It has been proposed^42^ that the calcium level represents an important intracellular messenger that initiates and regulates the apoptotic process^43^. Thus, the increase in intracellular calcium, also observed in this study, could lead to cell death. The time kill-kinetics (Fig. 2b) as compared to time depended depolarization (Fig. 4a) indicate that the change of potential membrane precedes cellular death. These is confirmed by the increase of intracellular calcium concentration (Fig. 4b). Thus we identify for the first time membrane depolarization and intracellular calcium levels as primary events in bacterial death.

### Induction of ROS and glutathione depletion in *K. pneumoniae* and *E. roggenkampii* cells exposed to argirium-SUNCs

**Figure 5:**
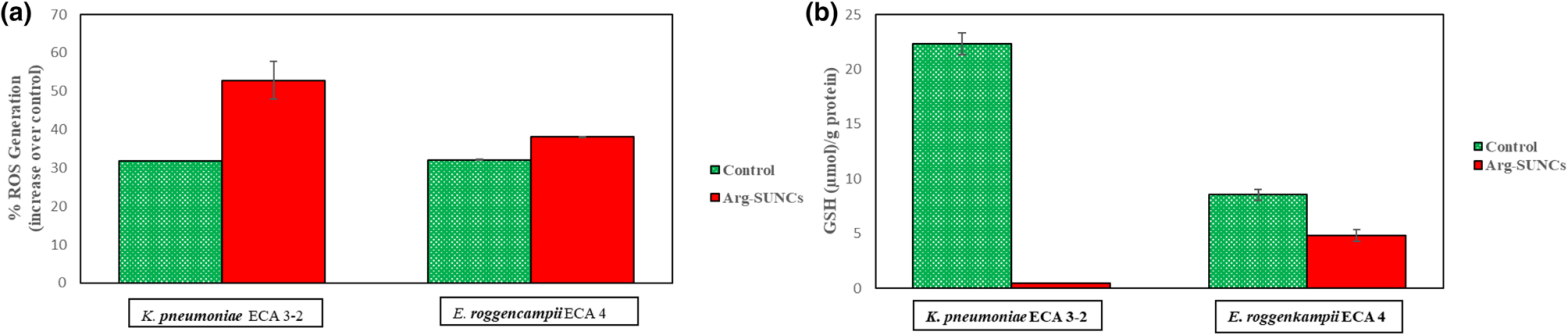
Analysis of the intracellular stress response to exposure from Arg-SUNCs in cell untreated (green-control), treated (red) with 5 ppm of Arg-SUNCs. (**a**) Increase indicate oxidative damage (ROS). (**b**) Intracellular glutathione levels (GSH). The data represents the average standard deviation.

In this part of the work we focused on intracellular stress mechanism observed. It is well know the toxicity of the nanomaterials is in part due to an increase in intracellular oxidative stress caused by the increase of ROS such as hydroxyl radicals (OH∙), superoxide ions (O_2_), hydrogen peroxide (H_2_O_2_), and hydroperoxyl radicals, which in turn induce a damage to proteins, lipids and nucleic acids and consequently the bacteria death. As observed in Fig. 5a, a significant increase in intracellular ROS levels was revealed after the exposure of bacteria to 5 ppm of SUNCs for 2 h. In particular, the most resistant strain *E. roggenkampii* ECA4 showed the lower ROS accumulation (39%) while *K. pneumoniae* ECA3-2 the most sensible strain, showed major ROS accumulation (53%), when compared to the control. In order to evidence if ROS generation caused by the Arg-SUNCs stress, also induced changes in glutathione (GSH) levels (Fig. 5b), we measured the intracellular GSH. This compound is regularly found at high concentrations, in the reduced form, in order to maintain redox levels inside the cell and protect it from the oxidative stress^44^. The result depicted in Fig. 5b shown that depletion of GSH by *E. roggenkampii* ECA 4, was lesser (near 43%) than those of K*. pneumoniae* ECA3-2 in which we don’t detect GSH, suggesting that this strain activated the defense mechanisms to counteract environmental changes due to the presence of Argirium® SUNCs. Some researchers have been demonstrated that bacteria exposed to silver nanoparticles exhibit increased ROS formation. In similar way, Yuan et al. suggested that *Pseudomonas aeruginosa* and *Staphylococcus aureus* cells treated with 2 and 4 ppm of silver nanoparticles respectively, exhibit increased ROS formation, decreased GSH levels, increased GST enzyme activity, and decreased cell viability. Thus, we could hypothesize that the depletion of GSH levels in cells subjected to ARGIRIUM-SUNCs was due to an excessive production of intracellular ROS that slowed the defense system and led to oxidative stress and a loss of cell viability. In contrast to our study, did not report ROS generation in *E. coli* and *P. aeruginosa* cells treated with silver nanoparticles. However, the authors reported that the nanoparticles were synthetized as protein (casein)-coated colloidal AgNP, and prepared at 2 mM (215.7 mg Ag/L, 20 mL) in autoclaved deionized water and further stored in the dark at + 4 °C and diluited with buffer (MOPS-Tris buffer). These important differences in the chemical property and in the synthesis method respect ARGIRIUM-SUNc do not permit their comparison in terms of the biological effect.

### Changes in volatile organic compound (VOCs) induced during sublethal stress of ARGIRIUM-SUNCs

Bacteria in general, are continuously detecting and answering to surrounding environmental conditions, comprising the presence of toxic compounds. As a part of the bacteria metabolism, they produced volatile organic compounds (VOCs), which serve as chemical windows through which the fundamental information about the molecular basis of microbial activities is released, giving an information on the real-time metabolic changes that occur within the cell. Thus to test how E. *roggenkampii* ECA4 and K. *pneumoniae* ECA 3-2 strains responded to sub-lethal stress of ARGIRIUM-SUNCs in terms of volatilome, cells were grown in BHI and exposed to nanomaterials concentrations of 0, 0.625, and 1.25 mg/L respectively. To estimate the metabolic changes, the relative abundance of the data of each VOC detected by SPME–GC–MS, they were normalized by subtracting those of the non-inoculated medium. The VOCs of the E. *roggenkampii* ECA4 and K. *pneumoniae* ECA 3-2 controls in liquid culture were very different. In fact, while we detected in the headspace of the samples inoculated with K. *pneumoniae* ECA 3-2 24 compounds, in those inoculated with E. *roggenkampii* ECA4 we detected only 16 compounds. In addition, seven compounds were unique for K. pneumoniae ECA 3-2, specifically: 2-butanone, 2-heptanone, 2 pentadecanone, 1 hexanol, 1-nonanol, 2-undecanol, and 2-tridecanol. Instead for *E. roggenkampii* 4, were 3-hydroxy Butanal, 2-Nonadecanone, and Octanoic acid ethyl ester.

**Figure 6:**
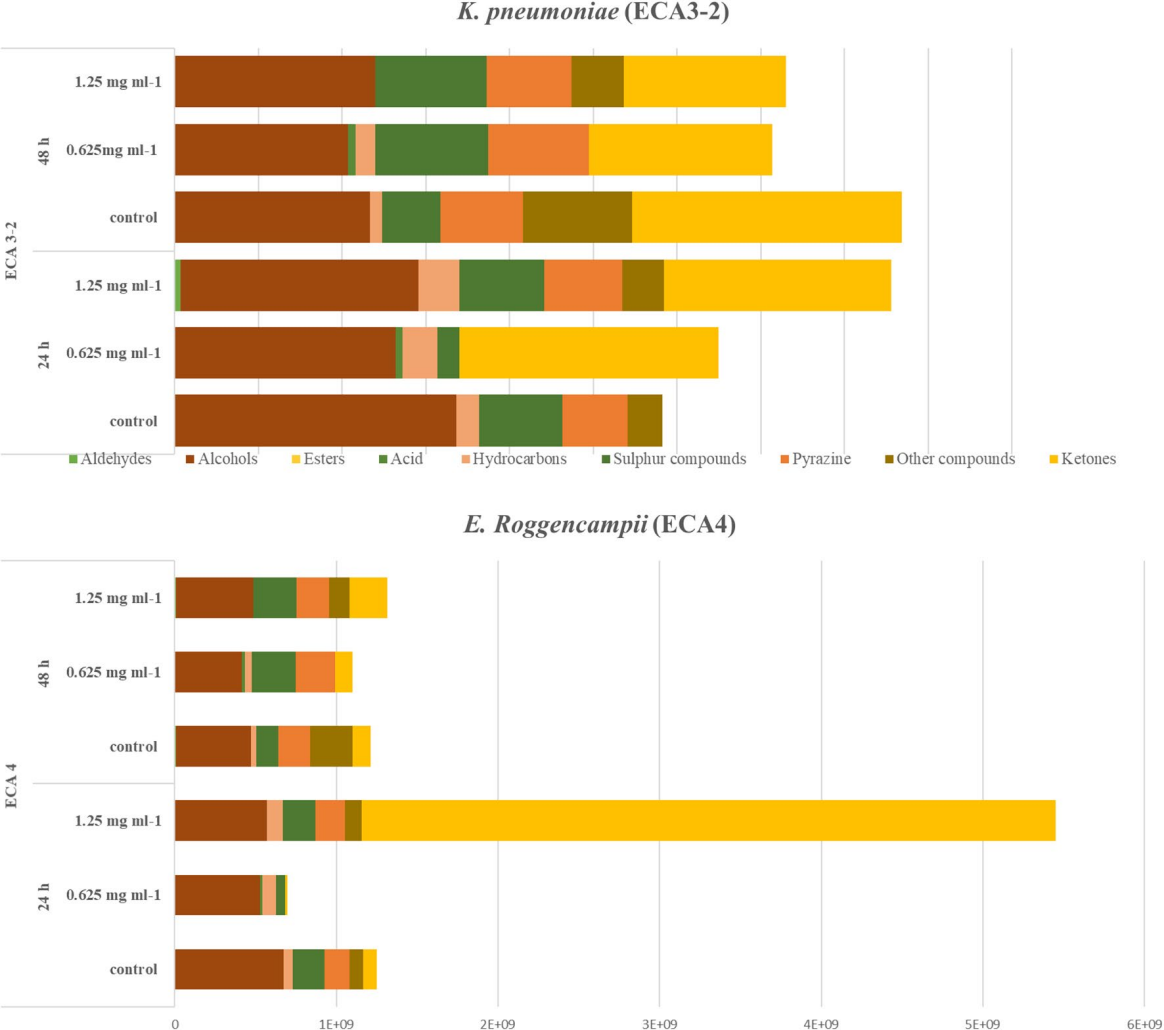
Volatile organic compounds (principal functional groups) produced by from *K. pneumoniae* (ECA3-2) and *E. roggenkampii* (ECA4), after and without the addition of Arg-SUNCs at 24 and 48 h of incubation.

Figure 6 shown the most important functional groups produced by the two strains with and without the addition of ARGIRIUM-SUNCs. In both of the species, ketones, alcohols and sulfur functionals groups were the most abundant compounds detected. It is to underline that some long-chain aliphatic alcohols (1-octanol, 1-decanol, and 1-dodecanol) produced by α- or β-oxidation of fatty acid derivatives, frequently are present in the *Enterobacteriaceae*. To our knowledge there are not reports about the *E. roggenkampii* VOCs in literature. The variation of the VOCs associated with the most relevant pathways in *E. roggenkampii* and *K. pneumoniae* strains after treatment with different concentration of ARGIRIUM-SUNCs are presented in Supplementing Materials. As observed most of the volatiles detected derived from amino acids and fatty acids metabolic pathways. From the comparison of the VOCs produced by treated and untreated strains, it was possible to evidence that acetic acid and tetradecane were the only particular compounds related to the presence of ARGIRIUM-SUNCs in both of the strains. Acetic acid, like other short-chain organic acids are the major side products of anaerobic metabolism of the carbohydrates by the *Enterobacteriaceae* and their high production could arrest the bacteria growth^45^. However in our experiments their concentration was not very high to have this effect. One possible explanation for the increase of acetate production could be related to a possible decrease in the energy status of the cells in presence of ARGIRIUM-SUNCs, that could be overcome using acetate to produce acetyl CoA which can be utilized in the Krebs cycle, thus shifting the metabolism from anaerobic to aerobic in *K. pneumoniae* ECA3-2. However, is worthy of note that 0.625 mg/L of ARGIRIUM-SUNCs, induces the generation of 2-ethyl-4-methyl-1-Pentanol in *K. pneumoniae* ECA3-2 samples while 2- pentadecanone, 1 decanol, 1-pentanol and 2-ethyl-5-methyl pyrazine was induced in *E. roggenkampii* ECA 4. In addition, it was observed an increase of 3-methyl-1-Butanol, that derived from the amino acid leucine, with the increase of ARGIRIUM-SUNCs concentration in both of the strains (Supplementary Materials). This fact demonstrated the induction of the Ehrlich pathway, in which aldehydes and subsequently alcohols are formed from the amino acids. Our findings are in contrast with those reported by Bos et al.^46,47^^9^, in which treatment with silver nanoparticles downregulated this metabolic pathway in *E. coli*. These differences could be attributed to the different nanoparticles concentration used as well as the measurements of the VOCs, which were obtained at different timepoints in the growth of bacteria. In the same way, the production of 2-Phenylethyl alcohol, which derives from l-phenylalanine was modulated by the ARGIRIUM-SUNCs concentration in both strains. The low concentration (0.625 mg/L) induced a reduction in their production, while high concentration (1.25 mg/L), induced a major emission of this compound. A positive correlation was observed between increase of ARGIRIUM-SUNCs and the emission of the methy ketones 2-butanone, 2-heptanone, 2-pentadecanone, which are formed from the fatty acids oxidation, in samples inoculated with *K. pneumoniae* ECA3-2 strain, and 1-dodecanol with *E. roggenkampii* ECA4 strain (Supplementary Materials). As previously discussed, the presence of ARGIRIUM-SUNCs induces the production of reactive oxygen species (ROS) which in turn, could trigger the oxidation of side chains in intracellular proteins, leading to their fragmentation and destabilization^47^. This fragmentation of proteins would have allowed a greater availability of amino acids, which probably led to an increase in the production of alcohols^48^ like 3-methyl-1-butanol. Various studies have shown that the accumulation of alcohols and aromatic compounds activate the response against its oxidative effects, which in turn cause damage at the intracellular level. The Free fatty acids peroxidation leads as a product alkanes, alkenes, alcohols, ketones, and aldehydes. (e.g., 2-ketones arising from the metabolism of fatty acids). It is known that in many organisms, ROS can oxidize unsaturated acyl chains integrated into membrane phospholipids. Therefore, the activation of this system allowed the continuous production of alcohols when *E. roggenkampii* ECA 4 was exposed to sub-lethal concentrations of ARGIRIUM-SUNCs (1.25 ppm), thanks to the continuous expulsion of these compounds from the cytoplasm to the external environment. Thus, we hypothesized that metabolic changes here reported emphasizes the role of ARGIRIUM-SUNCs as a direct or indirect transcriptional activator of pathway genes.

## Conclusion

It is well known that silver metal is able to coordinate electronegative atoms (oxygen) present in the functional groups of the biofilm (carboxyls, amides, esters of teichoic acid) and to replacing Ca^++^/Mg^++^/Cu^++^ ions. Silver reactions against aldehyde groups (Tollens reactions), disulfide bridges (–S–S–) and thiol groups that irreversibly bind metal ions are also known. Similarly, silver could inhibit surfactin function by preventing its interaction with calcium and magnesium ions necessary for its activity. We were interested in synthetizing a new formulation of silver that would amplify the already known reactivity of this metal.

Our ARGIRIUM-SUNCs formulation is more efficient for disrupting biofilm and bacterial toxicity than another’s commercial nanocomposite. The present data suggest that in addition to the well-known evidence that silver nanoparticles have effect on cellular metabolism, oxidative stress, toxicity of the ionic form and chelating and coordinating capacity of functional groups, other mechanisms should justify the greater efficacy of ARGIRIUM-SUNCs. We assume that our ultra nanoclusters being characterized by different Ag^0^/Ag^n+^ oxidative states, an high surface charge (zeta potential >  − 50 mV)^49^, a very small average size (nanocluster < 1.8 nm) and polygonal shape are effective to destructure the biofilm by interfering with the cross-links necessary for its three-dimensional structure. The biofilm disrupting occurs also in advanced growth situations. It is important to note that ARGIRIUM-SUNCs are very effective (0.6 ppm) against biofilm independently by their constitution. This supports the idea that different biofilm structures are characterized by similar cross-links which are the primary target of nanoparticles.

The Cross-linking with the genetic material of biofilm (eDNA) may alter cell–cell communication (quorum-sensing). Thus, interacting with the biofilm surfaces these nanoparticles may also modify the replication signals. Therefore the coordination capacity and its redox potential make the ARGIRIUM-SUNCs formulation extremely effective. Since bacterial biofilms and their constituents are essential for bacterial survival in contact with humans, its disrupting by ARGIRIUM-SUNCs activity represents a logical target for new clinical antibacterial treatments.

We observed that before cell death there is the phenomenon of the membrane polarization inversion. Contemporary the calcium channels resulted also altered. Thus, for the first time, we propose that the alteration of intracellular calcium concentrations and the membrane depolarization are primary events in bacterial death. Oxidative stress highlights how ROS and glutathione are defense strategies corresponding to different energetic metabolism as indicated by Volatilome results. ARGIRIUM-SUNc exhibited effective antibacterial properties at a very low nanoparticle concentration (0.6 ppm). In addition, when used in association with different chemotherapeutic molecules they also exhibited relevant synergistic properties. This suggest that SUNc’synthesis linked to traditional antibiotic could lead to new drugs potentially candidates to overcome the phenomenon of antibiotic resistance^50–52^.

## Supplementary Information

Supplementary Information.
