# Supplementary material for: The membrane depolarization and increase intracellular calcium level produced by silver nanoclusters are responsible for bacterial death
Source: Sci Rep. 2021 Nov 3;11:21557. doi: 10.1038/s41598-021-00545-7 (PMC8566483; doi:10.1038/s41598-021-00545-7)
Supplement: Supplementary file 1 — Supplementary Information. [file 41598_2021_545_MOESM1_ESM.docx]

**SUPPLEMENTARY MATERIAL**

**Table 1.** Antibiotic resistance of the strain used in this study, analysis performed

Kirby–Bauer and Vitek2 methods. From 1 to 20 Kirby - Bauer test - From 21-29 Vitek2

|  | **Antibiotic** | *Klebsiella pneumoniae* **ECA3-2** | *Klebsiella pneumoniae* **ECA20-3** | *Enterobacter hormaechei*  **ECA23-3** | *Enterobacter roggenkampii* **ECA4** |
| --- | --- | --- | --- | --- | --- |
| **1** | **NA Nalidixic Acid 30 μg** | S | S | S | S |
| **2** | **CN Gentamicin 10 μg** | S | S | S | S |
| **3** | **OL Oleandomycin 15 μg** | R | R | R | R |
| **4** | **ENR Enrofloxacin 5 μg** | S | S | S | S |
| **5** | **DA Clindamycin 2 μg** | R | R | R | R |
| **6** | **B Bacitracin 10 μg** | R | R | R | R |
| **7** | **LZD Linezolid 30 μg** | R | R | R | R |
| **8** | **TE Tetracycline 30 μg** | S | S | S | S |
| **9** | **UB Flumequine 30 μg** | S | S | S | S |
| **10** | **C Chloramphenicol 30 μg** | S | S | S | S |
| **11** | **E Erythromicin 15 μg** | R | R | R | R |
| **12** | **SP Spiramycin 100 μg** | R | R | R | R |
| **13** | **OX Oxacillin 5 μg** | R | R | R | R |
| **14** | **K Kanamicyn 30 μg** | S | S | S | S |
| **15** | **CIP Ciprofloxacin 5 μg** | S | S | S | S |
| **16** | **AK Amikacin 30 μg** | S | S | S | S |
| **17** | **KF Cephalothin 30 μg** | R | R | R | R |
| **18** | **AML Amoxycillin 10 μg** | R | R | R | R |
| **19** | **CXM Ceforoxime 30 μg** | R | S | S | S |
| **20** | **Piperacillin/Tazobactam** | S | S | S | S |
| **21** | **Cefotaxime** | S | S | S | S |
| **22** | **Ceftazidime** | S | S | S | S |
| **23** | **Cefepime** | S | S | S | S |
| **24** | **Ertapenem** | S | S | S | S |
| **25** | **Imipenem** | S | S | S | S |
| **26** | **Meropenem** | S | S | S | S |
| **27** | **Fosfomicin** | S | S | S | S |
| **28** | **Nitrofurantoin** | S | S | S | S |
| **29** | **Trimetoprim/Sulfametossazol** | S | S | S | S |

| **Compounds** | **Pathways** | ***K. pneumoniae* (ECA3-2)** | | | | ***E. roggenkampii* (ECA4)** | | | |
| --- | --- | --- | --- | --- | --- | --- | --- | --- | --- |
|  |  | **0.625 mg ml-1** | | **1.25 mg ml-1** | | **0.625 mg ml-1** | | **1.25 mg ml-1** | |
|  |  | **24 h** | **48 h** | **24 h** | **48 h** | **24 h** | **48 h** | **24 h** | **48 h** |
| 3-hydroxy Butanal | **F.A.O.** | n.r | n.r | +23.73 | n.r | n.r | n.r | n.r | +22.92 |
| 2- Butanone | **F.A.O.** | + 0.40 | + 0.53 | +0.49 | +1.10 | n.r | n.r | n.r | n.r |
| 2-Heptanone | **F.A.O.** | + 0.29 | n.r | +0.57 | +0.20 | n.r | n.r | n.r | n.r |
| 2-Nonadecanone | **F.A.O.** | n.r | n.r | n.r | n.r | abs | abs | abs | abs |
| 2-Pentadecanone | **F.A.O.** | + 0.37 | - 0.86 | +0.40 | +0.55 | n.r | +23.79 | +28.15 | +24.92 |
| Cyclopentadecanone | **F.A.O.** | abs | abs | + 0.25 | abs | +22.64 | +0.90 | +28.55 | abs |
| 2-Tridecanone | **F.A.O.** | + 0.44 | -0.89 | +0.44 | abs | -1.02 | -0.58 | + 5.45 | +1.73 |
| 2-Undecanone | **F.A.O.** | abs | - 0.74 | abs | +0.65 | abs | -0.30 | +4.66 | 1.01 |
| 2-Nonanone | **F.A.O.** | abs | -0.45 | -0.34 | +0.46 | abs | + 2.18 | +5.23 | abs |
| 4-methyl-2-Heptanone | **F.A.O.** | n.r | n.r | n.r | n.r | abs | abs | +7.07 | - 0.48 |
| Ethanol | **G.** | abs | abs | abs | abs | -0.21 | +27.09 | -0.21 | +26.71 |
| 1-Hexanol | **F.A.O.** | abs | abs | abs | abs | n.r | n.r | n.r | n.r |
| 1-Octanol | **F.A.O.** | abs | abs | -0.31 | abs | -0.32 | n.r | +4.25 | n.r |
| 1-Nonanol | **F.A.O.** | abs | abs | +0.71 | abs | n.r | n.r | n.r | n.r |
| 1-Decanol | **F.A.O.** | -7.02 | -3.67 | -0.14 | abs | abs | abs | +7.49 | n.r |
| 1-Undecanol | **F.A.O.** | abs | abs | abs | abs | n.r | n.r | n.r | n.r |
| 2-Undecanol | **F.A.O.** | abs | abs | abs | -0.16 | n.r | n.r | n.r | n.r |
| 1-Dodecanol | **F.A.O.** | - 1.14 | + 6.15 | abs | 3.81 | +1.02 | +24.36 | +1.56 | +26.62 |
| 2-Tridecanol | **F.A.O.** | abs | abs | +23.27 | abs | n.r | n.r | n.r | n.r |
| 3-methyl-1-Butanol | **E.P.** | +0.57 | +0.61 | +0.57 | 1.09 | +0.92 | +0.92 | +2.68 | +0.84 |
| 2-ethyl-4-methyl-1-Pentanol | **F.A.O.** | +24.24 | +23.45 | n.r | n.r | n.r | n.r | n.r | n.r |
| 2-Phenylethyl alcohol | **E.P.** | - 0.63 | - 0.39 | +0.54 | +0.74 | -0.77 | - 0.04 | 2.79 | - 0.65 |
| Cyclobutanol | **F.A.O.** | +22.33 | n.r | n.r | n.r | n.r | abs | n.r | abs |
| Octanoic acid. ethyl ester | **F.A.O.** | n.r | n.r | n.r | n.r | abs | abs | +5.50 | abs |
| Acetic acid | **G.** | +23.96 | +24.15 | n.r | n.r | +20.31 | 23.47 | +26.39 | +20.59 |
| Tetradecane | **F.A.O.** | n.r | n.r | +21.06 | n.r | +20.33 | n.r | +20.07 | n.r |
| 5-ethyl-2.2.3-trimethyl-heptane | **F.A.O.** | n.r | n.r | n.r | n.r | abs | abs | +3.87 | abs |
| 2.2.4.6.6.pentamethylheptane | **F.A.O.** | +0.61 | +0.61 | 0.80 | abs | n.r | n.r | n.r | 25.09 |
| Dimethyl disulfide | **M.C.** | - 1.83 | +1.06 | 0.004 | 1.04 | -1 | abs | 3 | +0.3 |
| Dimethyl trisulfide | **M.C.** | abs | abs | + 3.99 | abs | n.r | n.r | n.r | n.r |
| 2.5-dimethylPyrazine | **A. M.** | abs | +0.19 | +0.27 | -0.08 | +0.43 | - 0.27 | +3.91 | - 0.18 |
| 2-ethyl-5-methylPyrazine | **A. M.** | n.r | +23.98 | n.r | 23.95 | +22.96 | - 0.15 | abs | abs |
| Non identified (R.T) | **-** | abs | abs | 1 | abs | abs | 24,94 | abs | 25,45 |
| Non identified (R.T) | **-** | abs | n.r | -0,22 | -0,74 | 25,18 | n.r | 27,54 | n.r |
| abs = values absent after ARGIRIUM-SUNCs addition,  n.r = values non registered,  Positive symbol (+) indicate the values of metabolites are increases after treatment with ARGIRIUM-SUNCs,  Negative symbol (-) indicate the values of metabolites are decreases after treatment with ARGIRIUM-SUNCs,,  Cells red coloured with positive values (+) indicate that the metabolites are produced only after ARGIRIUM-SUNCs, addition.  Metabolic pathways of metabolites production: **F.A.O**.: Fatty Acids Oxidation, **G.:** Glycolysis, **E.P.:** Ehrlich Pathway, **M.C.:** Methionine Catabolism, **A.M.:** Aminoacids Metabolism. | | | | | | | | | |

**Table 2.** Altered volatile organic compounds (VOCs) and addressed bacterial metabolic pathways, where numbers inside boxes display the base 2 logarithm of fold-change calculated with respect to the response in untreated strain


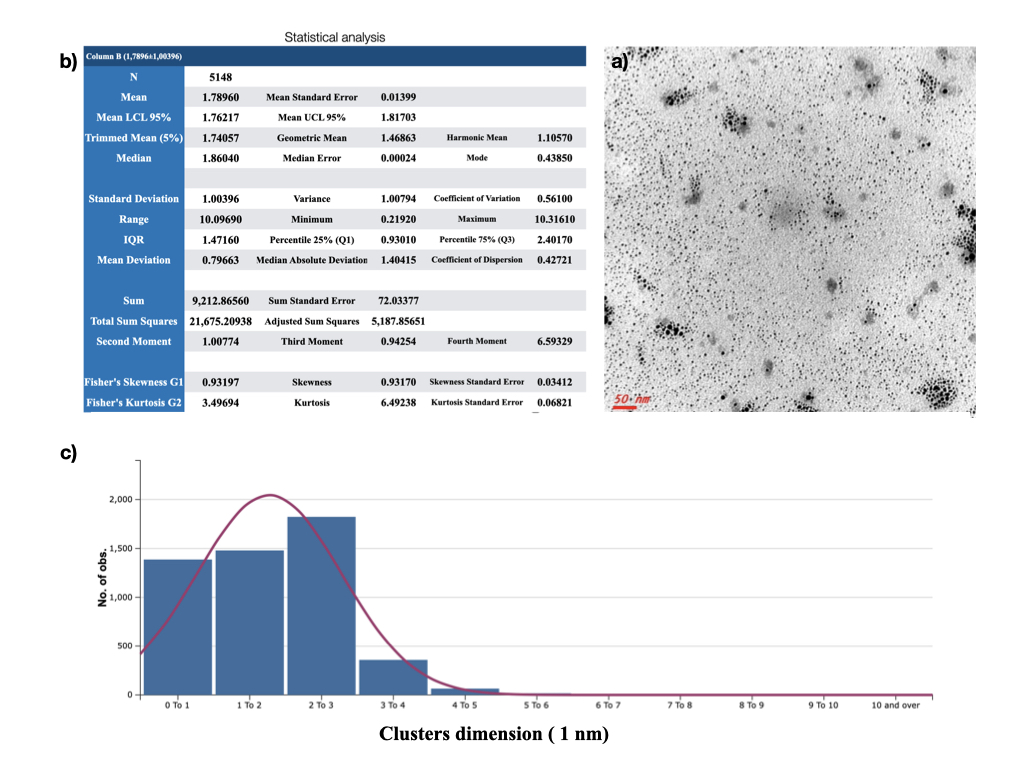


**Fig. 1S** To better quantify the size of nanoparticles showed in Fig.1a in the text we have performed the Statistical analysis of TEM results:

a) TEM image of filtered SUNc in ultra-pure water (0,22𝛍m) corresponding to Fig. 1a in the text (scale bar 50 nm)

b) Statistical analysis of TEM image (software used StatPlus-2 and ImaJ [1.8.0_172](https://wsr.imagej.net/distros/osx/ij153-osx-java8.zip) )

c) Histogram of the statistical size distribution.
